# Excised Knees

**Published:** 1859-07

**Authors:** 


Excised Knees.—A most curious and novel sight occurred the other night, at the London Medical Society, which took my fancy very much. Mr. P. C. Price, a young surgeon of great promise, a protege of my friend Mr. Ferguson, and who has at the same time performed most of the capital operations, read a paper on some of the causes of failure following the operation of excision of the knee-joint, in which he most ably considered the subject, and very satisfactorily showed that the want of a successful issue depended upon circumstances which might have occurred had amputation been performed. At the conclusion of his paper, some ten or more male and female persons walked into the room, each of whom had undergone resection of one of their knees, and who were living proofs of the value of a limb without a joint. One lad could walk his fourteen miles a day, without inconvenience or fatigue; all were in excellent health. The sight was rather amusing, too; for both males and females had their knees exposed whilst walking up and down to show their anarthrodial agility. Mr. Price is engaged in the preparation of a treatise on excision of the knee, which will be copiously illustrated, and at the same time will contain an account of every operation that has been done, up to the year 1858.—*Montreal Chronicle.*

